# Evaluation of a shared decision-making strategy with online decision aids in surgical and orthopaedic practice: study protocol for the E-valuAID, a multicentre study with a stepped-wedge design

**DOI:** 10.1186/s12911-021-01467-0

**Published:** 2021-03-29

**Authors:** Floris M. Thunnissen, Bernhard W. Schreurs, Carmen S. S. Latenstein, Marjan J. Meinders, Eddy M. Adang, Glyn Elwyn, Doeke Boersma, Bas Bosmans, Koop Bosscha, Bastiaan L. Ginsel, Eric J. Hazebroek, Jeroen J. Nieuwenhuis, Maarten Staarink, Dries Verhallen, Marc L. Wagener, Femke Atsma, Philip R. de Reuver

**Affiliations:** 1 Department of Surgery, Radboud University Medical Centre, P.O. Box 9101, 6500 HB Nijmegen, The Netherlands; 2 Department of Orthopaedics, Radboud University Medical Centre, P.O. Box 9101, 6500 HB Nijmegen, The Netherlands; 3 Radboud Institute for Health Sciences, IQ Healthcare, Radboud University Medical Centre, P.O. Box 9101, 6500 HB Nijmegen, The Netherlands; 4 Department for Health Evidence, Radboud University Medical Centre, P.O. Box 9101, 6500 HB Nijmegen, The Netherlands; 5 Department of Surgery, Jeroen Bosch Hospital, ‘s Hertogenbosch, The Netherlands; 6 Department of Orthopaedics, Slingeland Hospital, Doetinchem, The Netherlands; 7 Department of Orthopaedics, Queen Beatrix Hospital, Winterswijk, The Netherlands; 8 Department of Surgery, Rijnstate Hospital, Arnhem, The Netherlands; 9 Department of Orthopaedics, VieCuri Hospital, Venray, The Netherlands; 10 Department of Surgery, Van Weel Bethesda Hospital, Dirksland, The Netherlands; 11 Department of Value-Based Health Care, St. Anna Hospital, Geldrop, The Netherlands; 12 Department of Orthopaedics, Rijnstate Hospital, Arnhem, The Netherlands

**Keywords:** Shared decision-making, Decision aids, Cholecystolithiasis, Inguinal hernia, Knee osteoarthritis, Hip osteoarthritis

## Abstract

**Background:**

Inguinal hernia repair, gallbladder removal, and knee- and hip replacements are the most commonly performed surgical procedures, but all are subject to practice variation and variable patient-reported outcomes. Shared decision-making (SDM) has the potential to reduce surgery rates and increase patient satisfaction. This study aims to evaluate the effectiveness of an SDM strategy with online decision aids for surgical and orthopaedic practice in terms of impact on surgery rates, patient-reported outcomes, and cost-effectiveness.

**Methods:**

The E-valuAID-study is designed as a multicentre, non-randomized stepped-wedge study in patients with an inguinal hernia, gallstones, knee or hip osteoarthritis in six surgical and six orthopaedic departments. The primary outcome is the surgery rate before and after implementation of the SDM strategy. Secondary outcomes are patient-reported outcomes and cost-effectiveness. Patients in the usual care cluster prior to implementation of the SDM strategy will be treated in accordance with the best available clinical evidence, physician’s knowledge and preference and the patient’s preference. The intervention consists of the implementation of the SDM strategy and provision of disease-specific online decision aids. Decision aids will be provided to the patients before the consultation in which treatment decision is made. During this consultation, treatment preferences are discussed, and the final treatment decision is confirmed. Surgery rates will be extracted from hospital files. Secondary outcomes will be evaluated using questionnaires, at baseline, 3 and 6 months.

**Discussion:**

The E-valuAID-study will examine the cost-effectiveness of an SDM strategy with online decision aids in patients with an inguinal hernia, gallstones, knee or hip osteoarthritis. This study will show whether decision aids reduce operation rates while improving patient-reported outcomes. We hypothesize that the SDM strategy will lead to lower surgery rates, better patient-reported outcomes, and be cost-effective.

*Trial registration*: The Netherlands Trial Register, Trial NL8318, registered 22 January 2020. URL: https://www.trialregister.nl/trial/8318.

## Background

Inguinal hernia repair, gallbladder removal, and knee- and hip replacement are routine and elective surgeries. While international guidelines advocate surgery in the majority of patients, a wait-and-see policy is justified in selected cases [1–3]. Although surgery is associated with quick symptom relief, long term outcomes are sometimes less favourable [4–14]. Because of the elective nature of these interventions, treatment decision depends on multiple factors, such as the severity of symptoms, organizational structures, and physicians’ and patients’ preferences [15]. Some of these factors have a role in inappropriate care, unwarranted practice variation, and higher costs [16, 17].

Shared decision-making (SDM) leads to more appropriate care [18–20]. Allowing patients to have a more active role in their treatment decision results in a better consultation [17]. SDM balances empirical evidence, professional’s expertise and patient’s values [21]. In the case of SDM, clinicians need to provide patients with high-quality and balanced information. Clinicians need to assess the patients’ values and preferences, and assist and empower patients in their process of decision-making [22, 23]. To support the SDM process, decision aids (DAs) are used. DAs are tools providing evidence-based information on the different treatment options to improve the disease-specific knowledge of the patient, while exploring patient values [24–26]. In general, the use of DAs leads to better informed patients, who are able to select a choice of treatment based on accurate risk perceptions, who can make better value-congruent choices and have less decisional conflict [25, 27]. The possibility that patients will opt for less invasive treatment choices will lower the costs and are potential benefits of SDM from a patient and societal perspective [25].

The objective of this study is to evaluate the effect of an SDM strategy by using online DAs, specifically designed for patients who are eligible for inguinal hernia repair, gallbladder removal, knee- or hip replacement. This study will compare the SDM strategy to usual care, in terms of surgery rates, patient-reported outcomes, and cost-effectiveness. We hypothesize that the SDM strategy will reduce surgery rates, while patient-reported outcomes improve. The outcome of the E-valuAID-study will clarify if SDM with the use of DAs is beneficial for the patient and our healthcare system.

## Methods

The study protocol is designed in accordance with the CONsolidated Standards Of Reporting Trials (CONSORT) extension for stepped-wedge cluster randomized trials [28]. The study has been registered in the Netherlands National Trial Register (NTR) on the 22nd of January 2020 (Trial: NL8318, https://www.trialregister.nl/trial/8318).

### Design

**Fig. 1:**
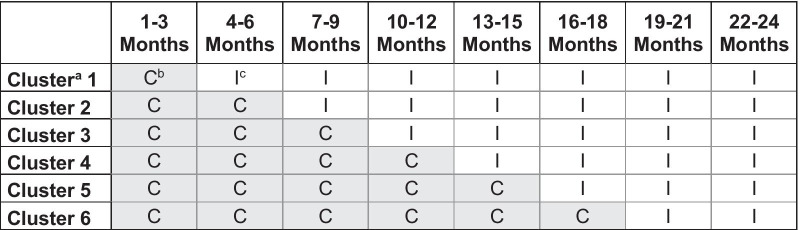
Stepped-wedge design on hospital level (primary outcome, surgery rates). ^a^A cluster consists of an outpatient clinic of surgery and an outpatient clinic of orthopaedics. ^b^C = Control (usual care). ^c^I = Intervention (SDM strategy with online DAs)

The E-valuAID is a multicentre, non-randomized stepped-wedge study in patients with an inguinal hernia, gallstones, knee- or hip osteoarthritis. The DAs will be implemented stepwise in six surgical and six orthopaedic departments in the Netherlands. Departments implement the DAs at different timepoints and will have different control and intervention periods. Therefore, the stepped-wedge design allows for comparisons within and between departments, and comparisons over time (Fig. 1). Three additional control hospitals will be included to investigate time trends in surgery rates for both surgical and orthopaedic departments across the entire study, without implementing DAs. Data collection began on July 1, 2020, and each centre will include consecutive eligible patients for two years (Fig. 1).

### Study population

The study aims to include all patients above the age of 18 who are referred to the outpatient clinic of participating departments, with one of the following conditions: an inguinal hernia, abdominal pain and ultrasound proven gallstones, knee- or hip osteoarthritis. Eligibility criteria are presented in Table 1. The secondary outcomes will be assessed in all eligible patients who agree to fill out the questionnaires.

**Table 1:** Eligibility criteria

Inclusion criteria	Age ≥ 18 years One of the following conditions: inguinal hernia, gallstones, knee- or hip osteoarthritis Access to the internet Sufficient command of the Dutch language to complete decision aids and fill out questionnaires
General exclusion criteria	Current treatment for malignancy Expected lifespan < 12 months Pregnancy
Condition related exclusion criteria	
Inguinal hernia	Recurrent or complicated inguinal hernia (e.g. incarcerated hernia, strangulation, ileus)
Gallstones	Complicated gallstone disease (e.g. cholecystitis, choledocholithiasis, biliary pancreatitis)
Knee- or hip osteoarthritis	Early-stage osteoarthritis Earlier arthroplasty

### Patient recruitment

Screening for eligibility will be performed by the local nurse practitioner, surgeon, or involved study researcher. Patients will be provided with information about the study, asked to participate, and informed consent will be obtained.

### Outcomes

**Fig. 2:**
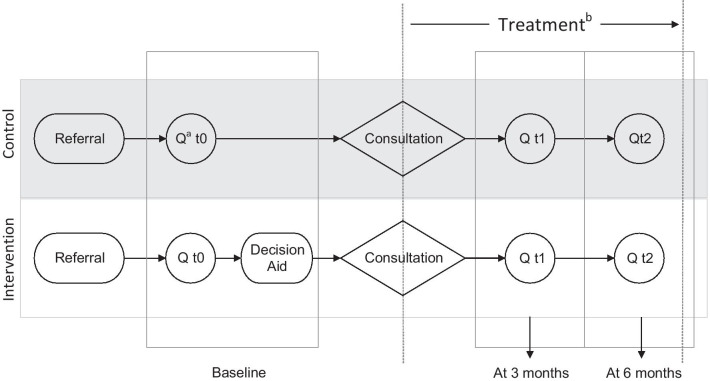
Clinical pathway and data collection timeline on patient level (secondary outcomes). ^a^Q: Questionnaire. ^b^The definitive treatment, conservative or surgery, will be assessed 6 months after inclusion

The primary outcome of the E-valuAID-study is the percentage of patients undergoing surgery within 6 months after first presentation at the outpatient clinic. Secondary outcomes are patient-reported outcomes and cost-effectiveness of the SDM strategy. Figure 2 provides an overview of the clinical pathway, data collection, and the timeline in which the DA and questionnaires are provided.

### Control group

In the control group, that is before implementation of the SDM strategy, all participating departments will reach treatment decision according to usual care. Usual care consists of treatment decisions based on clinical information (i.e. severity of symptoms, medical history, physical examination, and medical imaging), the physician’s knowledge and experience, as well as both the patient’s and physician’s preferences.

### Intervention group

At a pre-determined timepoint illustrated in Fig. 1, the SDM strategy will be implemented at department level and the department moves to the intervention phase. Details on the implementation are described below. In the intervention phase, an online DA is provided to each patient before consultation in which treatment decision is made (Fig. 2).

The condition-specific DAs are developed in accordance with the International Patient Decision Aid Standards (IPDAS) [24, 26] and in collaboration with the Dutch Society for Surgery (Dutch: NVvH, Nederlandse Vereniging voor Heelkunde), Dutch Orthopaedic Association (Dutch: NOV, Nederlandse Orthopaedische Vereniging), the Netherlands Patients Federation (Dutch: Patiëntenfederatie Nederland, PFN) and medical specialists. All DAs are approved by the Dutch Foundation of Easy Reading, who judged that the content is understandable for most people.

The clinical content of the DAs directly reflects current Dutch evidence-based guidelines on the treatment of inguinal hernia, gallstones, knee- or hip osteoarthritis [2, 3, 29, 30].

The DAs consist of four steps:

1. Information about the condition and treatment options (surgery versus wait and see).
2. A table which displays benefits, harms and probabilities of outcomes of treatment options, plus a knowledge questionnaire to remind about the most important information.
3. Value clarification exercises which help the patient to clarify what matters most to them to decide on a treatment preference.
4. Reflection on responses and indicating treatment preference.

**Fig. 3:**
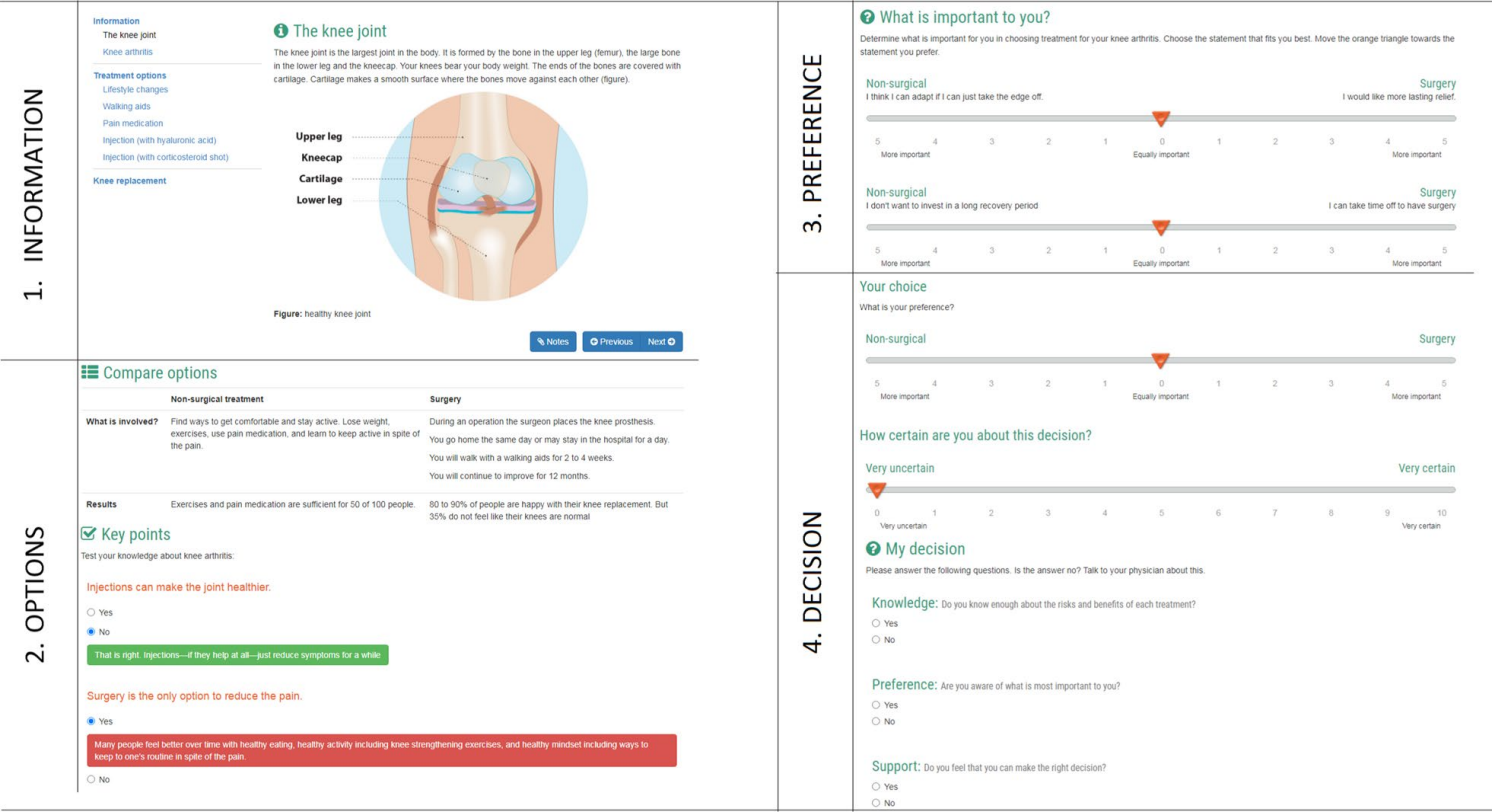
Summarized example of the DA for knee osteoarthritis, with the four corresponding steps

The layout of the DAs is displayed in Fig. 3.

Step three and four of the online DA contain a survey to assess the following information: patient’s values, patient’s treatment preference, and certainty about this preference. The patient’s values which are assessed are: concerns about surgery, concerns about complications, impact on daily activities, and experienced discomfort and pain due to the condition. Patients report their preference and their values on a slider ranging from − 5 to 5, as illustrated in Fig. 3. A score of -5 reflects the preference for watchful waiting, and a score of 5 reflects the preference for surgery. A patient’s certainty about their treatment preference is surveyed with a similar slider, ranging from 0 to 10.

### Implementation

Implementation of the SDM strategy is in accordance with a standardized protocol, which consists of four steps: (1) preparation, (2) instruction, (3) implementation of the DA and (4) evaluation.

1. During preparation, a local project group is assigned, including a physician, nurse practitioner, front- and back-office support, representative of the communication- and quality control department and the local project leader of the study. The group is responsible for setting goals, defining key results and identifying the clinical pathway of patients.
2. The instruction consists of 3 workshops for healthcare providers and desk employees to inform about SDM and to give instruction on the use of the DAs.
3. Implementation of the online DA executed by the local project group under supervision of the project leader of the study.
4. During evaluation, all stakeholders from the participating department are asked about their experiences with the SDM strategy.

Implementation at different departments will be performed after time intervals of 3 months and within a time window of 2 weeks. After 18 months, all departments will work according to the SDM strategy, patient inclusion will continue for another 6 months. For an overview of the stepped-wedge design and implementation scheme, see Fig. 1.

### Data collection

The primary outcome, the surgery rates within 6 months after first presentation at the outpatient clinic, will be retrieved from the electronic medical record (EMR) system of each hospital. Secondary outcomes will be retrieved from the EMR system, online questionnaires, and the patient-reported information assessed by the online DA.

EMRs will be used to extract treatment choice (surgery or conservative treatment) and patient characteristics (gender, age, weight, and height). Questionnaires will be administered online to assess secondary outcomes. All eligible patients will be asked to fill-out these questionnaires at three timepoints: before consultation in which treatment decision is made (baseline), and 3 and 6 months after baseline. Informed consent will be obtained before first questionnaire and to avoid contamination, the first questionnaire will be filled out before patients access the DA during the intervention phase.

A detailed overview of the questionnaires is summarized in Table 2. Briefly, the following questionnaires will be used: The CollaboRATE to assess the quality of the decision-making process from the patient perspective [31], the EuroQol-5D-5L (EQ-5D-5L) for generic quality of life [32], the EuraHS for patients with an inguinal hernia [33], the Gallstone Symptom list (GSL) for patients with gallstones [34], the Knee injury and Osteoarthritis Outcome Score—Physical function Short form (KOOS-PS) for patients with knee osteoarthritis [35], the Hip disability and Osteoarthritis Outcome Score—Physical function Short form (HOOS-PS) for patients with hip osteoarthritis [36], and the Short Form—Health and Labour Questionnaire (SF-HLQ) for the consequences of health problems [37]. Questionnaires will be provided online. After 1 week, patients will receive reminders by email up to three times to complete the questionnaires.

**Table 2:** Overview of questionnaire

Questionnaire	Outcome measure	Details	Baseline	3 months	6 months
CollaboRATE [31]	Level of SDM	A 3-item questionnaire measuring the experienced level of SDM	–	x	–
EuroQol-5D-5L (EQ-5D-5L) [32]	Quality of life	A 6-item questionnaire across 5 domains: mobility, self-care, usual activities, pain/discomfort and anxiety/depression. Each dimension has 5 levels: no problems, slight problems, moderate problems, severe problems and extreme problems. The questionnaire is scored with a 1-digit number that expresses the level selected for that dimension. The digits for the five dimensions can be combined into a 5-digit number that describes the patient’s health state. The last question is a VAS-score (range: 0–100) representing the patient’s self-rated health with 100 being ‘The best health you can imagine’ and 0 being ‘The worst health you can imagine’	x	x	x
Gallstone Symptom List (GSL) [34]	Disease related complaints	A 6-item questionnaire. This specific pain score was designed and previously used in a large cohort of patients in the United Kingdom. This questionnaire was designed to asses symptoms associated with symptomatic cholecystolithiasis	x^a^	x^a^	x^a^
EuraHS [33]	Disease related complaints	A 9-item questionnaire across 3 domains: pain, restriction of activities, and cosmetic discomfort. The questionnaire is scored on an 11-point scale from 0–10, total score ranges from 0–90 with low scores being favourable outcomes	x^a^	x^a^	x^a^
Knee injury and Osteoarthritis Outcome Score (KOOS-PS) [35]	Disease related complaints	A 7-item questionnaire about physical functioning of patients with knee complaints, experienced in the last week. The questionnaire is scored on a 5-points Likert scale (0–4) where 0 is no difficulty experienced and 4 is extreme difficulty	x^a^	x^a^	x^a^
Hip disability and Osteoarthritis Outcome Score (HOOS-PS) [36]	Disease related complaints	A 5-item questionnaire about physical functioning of patients with hip complaints, experienced in the last week. The questionnaire is scored on a 5-points Likert scale (0–4) where 0 is no difficulty experienced and 4 is extreme difficulty	x^a^	x^a^	x^a^
Short Form Health and Labour Questionnaire (SF-HLQ) [37]	Consequences on employment	This questionnaire concerns the consequences of health problems for employment in a paid job and for unpaid work (e.g. household chores). These questions pertain to the period covering the past month. Health problems refer both to your physical and emotional problems	x	x	x

^a^Patients only receive one of these questionnaires per timepoint, depending on patient's condition

### Sample size

Sample size calculations are based on differences in operation rate before and after the introduction of SDM. The sample size calculation is based on the formulas for a stepped-wedge design as proposed by Woertman et al. [38] Based on existing literature, the following reduction in surgery rate per condition is assumed: 10% for inguinal hernia, 10% gallstone disease, 5% for knee osteoarthritis, and 7% for hip osteoarthritis [39–43].

With the assumptions of a ß of 0.8, an a of 0.05 (two-sided) and an intraclass correlation of 0.10, the estimated sample sizes for inguinal hernia, gallstone disease, knee- and hip osteoarthritis are 50, 45, 120, and 95 patients per department per 3 months, respectively. Total estimated sample sizes for inguinal hernia, gallstone disease, knee- and hip osteoarthritis are 2400, 2160, 5760, and 4560 patients, respectively.

### Effect evaluation

Surgery rates will be calculated at department level each 3-month period and evaluated over time. Comparisons of surgery rates will be made before and after implementation of the SDM strategy.

Secondary outcomes will be compared between patients included before and after implementation of the SDM strategy.

### Cost-effectiveness

The economic evaluation will be undertaken alongside the clinical study, as cost-effectiveness analysis (CEA) with the costs per patient at 6 months follow-up. Additionally, a cost-utility analysis (CUA) will be performed with the costs per quality-adjusted life-year (QALY) as outcome. Both analyses will be performed from a societal perspective, and the time horizon is set at 6 months.

Both direct and indirect medical costs will be considered. These direct and indirect medical costs include: costs of inpatient and outpatient hospital stay, major diagnostic and therapeutic procedures, consultations, costs of out-of-hospital care by general practitioner and allied healthcare providers, non-medical out-of-pocket expenses (over-the-counter medication, informal care), and indirect non-medical costs of production loss from sick leave. These costs will be determined with data from the EMR and with data from questionnaires. The EQ-5D-5L questionnaire will be used at different timepoints to generate health status scoring profiles over time, which will be transposed into health utilities using country specific population-based tariffs of time trade-off ratings of health states.

### Process evaluation

In a process evaluation we will assess facilitators and barriers that influence the implementation process and adoption of the SDM strategy, making use of existing frameworks for the implementation of SDM [44–46]. This evaluation will also yield more in-depth information about relevant contextual factors and experiences of patients and healthcare providers (i.e. physicians and nurses) with the SDM strategy in general and with the DAs more specific. For this purpose, we will organize focus groups with patients and healthcare providers.

### Statistical methods

Baseline characteristics will be described using descriptive statistics. If continuous, Student’s t-test will be used to calculate differences between clusters for normally distributed data. Mann–Whitney U-test will be used for skewed data.

Mixed models will be used to analyse the data from the stepped-wedge design and to account for clustering of patients within hospitals at different follow-up periods. We will analyse the effect of the intervention in comparison with usual care on the primary outcome surgery rates. We will adjust for confounding factors, such as age, sex, clinical parameters (e.g. comorbidity), and working and social activities. Furthermore, we will use mixed models to analyse the effect of the SDM strategy regarding the secondary outcomes and to account for clustering of repeated measurements within patients and clusters of patients within hospitals. In addition, we will include time as a covariate in order to investigate time trends. When applicable, we will test assumptions of normality and linearity, and interaction terms.

#### Safety monitoring and data access

Data will be captured and stored in an online registry (Castor EDC) during the control phase. During intervention phase, answers to questionnaires and in DAs will be stored in a second secured environment, which is password protected and can only be accessed by investigators. All data will be stored pseudonymized. Locking of data and code list will take place after the end of follow‐up of the last included patient, and data will be stored for 15 years. Data management will be performed in accordance with FAIR guiding principles [47].

#### Ethical consideration

The protocol of the study was registered under file number 2018-4815 and assessed by the Central Committee on Research Involving Human Subjects of Arnhem-Nijmegen (CCMO Arnhem-Nijmegen). The committee confirmed the Medical Research Involving Human Subjects Act (WMO) does not apply. Local ethics assessment committees of the participating hospitals are requested to approve the protocol as well.

## Discussion

The results of the E-valuAID will clarify if the implementation of an SDM strategy with online DAs in surgical- and orthopaedic practices reduces surgery rates and is cost-effective compared to usual care. This multicentre study with a stepped-wedge design will show the effect of an SDM strategy on surgery rates, patient-reported outcomes, and cost-effectiveness. We hypothesize that the application of DAs will result in lower surgery rates, while improving patient-reported outcomes.

### Rationale of the study design

In the present study we chose a pragmatic approach to investigate the effectiveness of SDM with an online DA. A recent systematic review shows that the stepped-wedge design is particularly used for practical and methodological purposes, which makes it well-suited for this study [48]. For example, as only a limited number of clinics start simultaneously, this design allows a thorough implementation of the intervention in each clinic. The implementation protocol requires a dedicated introduction of SDM to the users by providing information about the study and the online DA. Moreover, the design allows for evaluation of both the effect on surgery rates on a hospital level and outcomes on a patient level. An alternative design of a randomized trial on patient-level would have the potential pitfall of contamination of usual care by the intervention strategy, as it is conceivable that specialists transfer their SDM experience and methods to usual care. Cluster randomization of departments appeared not feasible during the explorative discussions with participating hospitals. They strongly preferred to start the implementation of the SDM strategy at a predetermined moment in time, and our logistic inability to implement randomly decided to refrain from cluster randomization. In the present design, the potential risk of selection bias, due to clinicians’ decision of not providing an SDM strategy is negligible, as all consecutive patients receive a DA before their first consultation.

### Current evidence for SDM in surgery

Although the effect of SDM and provision of DAs on patient satisfaction are promising [49, 50], there is a knowledge gap on the impact of SDM-instruments in terms of cost-effectiveness. Up to date, no trials are performed to evaluate the cost-effectiveness of DAs in patients with an inguinal hernia or gallstones. One study evaluated the impact of a DA consisting of a 50 min video and a booklet in 343 patients with knee- or hip osteoarthritis. This RCT showed DAs were cost-effective, with similar health outcomes [51]. Furthermore, Stacey et al. reviewed the literature in 2017, and they included four trials with 570 patients with knee or hip osteoarthritis [40, 41, 52, 53]. These studies assessed various effects of a DA: patient knowledge, the efficiency of decision-making, or wait times between screening and definitive choice, but not on cost-effectiveness. Only our retrospective evaluation on the effect of online DAs in patients with an inguinal hernia or cholecystolithiasis suggested a reduction in surgical interventions, but a cost-effectiveness analysis was lacking [39]. In the absence of existing evidence, it is clear that more knowledge on the benefits of SDM from patient and societal perspectives is necessary. The E-valuAID aims to solve the current void in the literature on the benefits of SDM and online DAs. Results of the present study as proposed here, will affect healthcare policy concerning decision-making in surgery for the coming decades.

In conclusion, SDM is eminently applicable in patients with an inguinal hernia, gallstones, knee- or hip osteoarthritis due to both a surgical and conservative treatment option. This study will show whether an SDM-strategy will lead to a reduced surgery rate and if future implementation is cost-effective.

## Data Availability

Not applicable.

## Abbreviations

CEA: Cost-effectiveness analysis
CUA: Cost-utility analysis
CONSORT: Consolidated standards of reporting trials
DA: Decision aid
EMR: Electronic medical record
EQ-5D-5L: EuroQol-5D-5L
GSL: Gallstone symptom list
HOOS-PS: Hip disability and Osteoarthritis Outcome Score—Physical function Short form
IPDAS: International patient decision aid standards
KOOS-PS: Knee injury and Osteoarthritis Outcome Score—Physical function Short form
NTR: Netherlands Trial Register
QALY: Quality-adjusted life-year
SDM: Shared decision-making
SF-HLQ: Short Form—Health and Labour Questionnaire
WMO: Wet Wetenschappelijk Medisch onderzoek, Medical research involving human subjects act
